# King's College Hospital Festival Dinner

**Published:** 1903-05-02

**Authors:** 


### KING'S COLLEGE HOSPITAL FESTIVAL DINNER.

THE Lord Chief Justice (Lord Alverstone) presided at the annual festival dinner of King's College Hospital on Tuesday, 28th ult. A large number of guests sat down, and the financial result was most satisfactory, £2,148 being announced as the outcome of the collection made, which is some £400 more than the sum so raised last year.

In proposing the toast of "Success to the Hospital," the Chairman said that there were many aspects from which one could claim support for the institution on whose behalf they were gathered. They might look at the long list of names of men of science and skill who had drawn their learning and experience from it, and in that respect King's College Hospital was second to none. They had a noble heritage, and they were anxious to maintain it. After enumerating many notable sons of the College who had passed away, he alluded to Lord Lister, who was still with them, and who was famous not only in Great Britain but throughout the whole civilised world. They might appeal for support, because of their great medical school, which they had shown they were determined to maintain in the front rank of the Metropolitan hospitals. But these were not the only grounds on which they could advocate their cause; nothing in fact touched him more than the great good done to the poor and suffering. What he admired in the reports of King's College Hospital was that although sometimes they were in great financial difficulties, yet they never hesitated to go forward with their work and so set an example of courageous conduct which might well be followed. If there was one thing more than another that should urge them to make sacrifices it was that the highest medical skill was given without pecuniary reward, men of the greatest eminence in their profession giving their services freely. According to their means therefore they ought to do something to assist to relieve suffering and pain by helping the hospital to greater efficiency. And they must remember also those honoured women who, as nurses, were living as noble lives as could be lived. If these made their sacrifices they could call on them to make theirs, and it was in that spirit he asked for their support. They knew that whether the amounts were large or small whatever they gave would be well and wisely expended.

Lord Dillon, the Chairman of the committee of management, responded to the toast. The question of whether the hospital was wanted in the neighbourhood in which it stood was, he said, a delicate one, but so long as they could see a list of patients such as that dealt with last year he thought there could be no doubt that they ought to stay where they were. Their present position had been associated with some of the greatest triumphs of medical science and he did not doubt the future would supply as many more. The neighbourhood, to parody Shakespeare, was suffering, not a sea change, but an L.C.C. change. The County Council was laying low many houses which should have been laid low years before; but the houses to be erected would be occupied by people who would require the same skill and attention as before. He knew this was a question on which there was a difference of opinion, but it seemed to him that if South London required hospitals, South London should provide them. He was certainly not in favour of emigration, and though some of their young men might go and help to found a new institution he should be sorry to see King's migrate to South London or anywhere else. They had lived where they were long enough to have acquired a name not lightly to be parted with or moved to some other locality. He deprecated interference from the Government or assistance from the County Council, but he thought they ought to receive some support from distant parts considering how many patients they received from them. He admitted that, from their cramped situation, it was impossible to expand, but he attached more importance to the quality of the work done than to an increased quantity. It was the way people were relieved, not the number, to which he attached most importance. In conclusion, he said hospitals must live on hope. They had been most fortunate in the past, and it would be a poor compliment to Providence if they did not trust Providence to honour their cheques in the future.

Mr. Charles Awdry, the Treasurer, read out the amounts received before and at the dinner, and proposed the Chairman's health. After which the proceedings terminated.

